# A novel anxiety-associated SNP identified in *LYNX2* (*LYPD1*) is associated with decreased protein binding to nicotinic acetylcholine receptors

**DOI:** 10.3389/fnbeh.2024.1347543

**Published:** 2024-12-23

**Authors:** Kristin R. Anderson, Wenpeng Cao, Hui Sun Lee, Mark A. Crenshaw, Talulla B. Palumbo, Ethan Fisher-Perez, Amanda DeGraaf, Peter Rogu, Maria A. Beatty, Gabrielle M. Gracias, Avani V. Pisapati, Katie Hoffman, Krystle J. McLaughlin, Almut Hupbach, Wonpil Im, X. Frank Zhang, Julie M. Miwa

**Affiliations:** ^1^Department of Biological Sciences, Lehigh University, Bethlehem, PA, United States; ^2^Department of Bioengineering, Lehigh University, Bethlehem, PA, United States; ^3^BS, Robert Wood Johnson Medical School, Rutgers, The State University of New Jersey, New Brunswick, NJ, United States; ^4^Department of Psychology, Lehigh University, Bethlehem, PA, United States

**Keywords:** *Lynx2*, *LYPD1*, nicotinic acetylcholine receptors, anxiety disorders, human psychology, human DNA analysis, atomic force microscopy

## Abstract

**Introduction:**

Anxiety disorders are among the most common mental illnesses in the US. An estimated 31.1% of U.S. adults experience any anxiety disorder at some time in their lives. Understanding some of the molecular underpinnings of anxiety could lead to improved treatments over current strategies focusing on symptom relief rather than root causes. One significant neurotransmitter system exerting control over anxiety is the nicotinic receptor subdivision of the cholinergic system. The murine *Lynx2* gene, encoding a protein modulator of nicotinic acetylcholine receptors, is expressed in anxiety-related neural circuitry in rodents and has been functionally associated with anxiety-like behavior.

**Methods:**

We examined variations in the human *LYNX2* (*LYPD1*) gene and their potential effects on anxiety levels in a cohort of 624 participants. Participants completed validated anxiety questionnaires (e.g., STICSA and STAI), which assessed both their current anxiety and their general tendency to experience anxiety. Possible functional alterations due to one such mutation was assessed through atomic force microscopy (AFM) and computational modeling.

**Results:**

We identified a previously unreported single nucleotide polymorphism (SNP) in the mature protein-coding region of *LYNX2* that was associated with significantly higher than normal anxiety scores. These elevated scores resembled those seen in patients clinically diagnosed with generalized anxiety disorder and panic disorder, although this genetically defined subpopulation did not typically report such diagnoses. Through computational modeling of the homopentameric α7 nicotinic receptor subtype and in vitro atomic force microscopy (AFM), we discovered that a specific LYNX2 SNP is linked to a reduced binding affinity between the LYNX2 protein and nAChRs, offering a potential functional explanation for the role that this mutation may play in anxiety.

**Discussion:**

A polymorphism in LYNX2, which codes for an inhibitory modulator of nicotinic acetylcholine receptors, has the potential to lead to sensitized nicotinic receptor activity in anxiety-related circuits. The LYNX2 protein has been shown to bind to multiple nicotinic acetylcholine receptor subtypes, including α4β2, α7, and α3β4 subtypes, each of which have been shown to be involved in affective behaviors. This work suggests that a subpopulation of individuals harboring a deleterious mutation in LYNX2 may predispose them to anxiety through abnormal nicotinic receptor control. In the future, this work may lead to the development of a biomarker for anxiety or a diagnostic tool for the early detection of individuals with susceptibility to anxiety.

## 1 Introduction

The stress response is an adaptive set of physiological and psychological changes that induces a response to situations perceived as threatening. If not regulated properly, such responses can manifest into a suite of anxiety, mood, and stress-related disorders. Anxiety disorders are the most prevalent mental disorders in the United States (Bandelow and Michaelis, 2015). Despite this, the exact etiology of such disorders is not fully understood; it likely stems from complex interactions between genetic, psychological, and environmental factors (Feder et al., 2009; Lissek et al., 2014; Duits et al., 2015). Current treatments are therefore largely focused on treating symptoms rather than addressing the biological structures most fundamental to the development of an anxiety disorder (Locke et al., 2015; Williams et al., 2017). This highlights an urgent need to understand the complex underpinnings of anxiety to inform targeted, effective, and accessible treatments.

There has been substantial research uncovering the role of specific genes in the development of anxiety-related disorders (Martin et al., 2009); however, to become clinically useful, more research is needed to determine how specific genetic variants alter the susceptibility or risk of an individual developing an anxiety disorder when combined with environmental stressors (Bystritsky et al., 2013). Additionally, understanding if specific genetic variants increase the risk for developing anxiety disorders can help identify biomarkers, ultimately leading to better diagnostic and preventative measures. Although animal models under controlled exposure have proven useful for uncovering the role of specific genes in various behaviors (Le-Niculescu et al., 2011), biological detection of anxiety disorders remains complex, and examination of the human genome is one way to gain more precise insight into their pathology and treatment.

Studies in animal models have uncovered the role of the *Lynx2 (Lypd1)* gene in anxiety-related behaviors. *Lynx2* null mutant mice exhibit anxiety-like behavior as measured by light–dark box, elevated plus and social interaction behavioral assays, as well as elevated fear conditioning responses (Tekinay et al., 2009). Murine *Lynx2* is expressed in regions with known involvement in anxiety circuitry, such as the basolateral amygdala, cingulate cortex, and medial prefrontal cortex, as well as in other regions such as the septum, hippocampus, striatum, pedunculopontine tegmental nucleus, peripheral neurons, and pontine nuclei (Sherafat et al., 2021; Dessaud et al., 2006; Tekinay et al., 2009) and in human brains, has been shown to be expressed in specialized neurons within the anterior cingulate cortex (Yang et al., 2015). As such, *Lynx2* has neuronal functions outside of anxiety-related behaviors, in addition to processes outside of the nervous system (Wang et al., 2021; Sugimori et al., 2024).

*LYNX2* is a member of the L*y6/uPAR* gene superfamily, many members of which encode small three-fingered fold receptor-binding proteins (Tsetlin, 2014). Well characterized members within this superfamily are secreted snake venom proteins such as *α*-bungarotoxin, which binds and inhibits nicotinic acetylcholine receptors (nAChRs). The LYNX2 protein also has been shown to bind to nAChRs but it contains a motif for a GPI-anchor allowing it to embed in the neuronal membrane as a peripheral membrane protein (Pisapati et al., 2021; Paramonov et al., 2020; Zaigraev et al., 2022). nAChRs are ligand-gated ion channels that respond to the natural neurotransmitter acetylcholine, as well as the drug of abuse nicotine, and are implicated in a wide number of processes, including anxiety, but also extend to cognition, addiction, attention to name a few (Mineur et al., 2023). The LYNX2 protein has been shown to bind to several nAChR subtypes, including α4β2 (Tekinay et al., 2009), α7, and α3α4 nAChRs (Pisapati et al., 2021). Some of the biophysical properties of the interaction of LYNX2 with nAChRs have been studied with α4β2 nAChRs, the most abundant receptor subtype in the brain. These properties include lower responsiveness to agonist (seen as a rightward shift in EC_50_), faster desensitization of agonist-induced receptor responses (Tekinay et al., 2009), inhibition of calcium fluxes through the receptor, and suppression of surface α4β2 receptors even upon nicotine exposure (Wu et al., 2015).

Animal studies have demonstrated that nAChRs, including the α7, α3β4 and α4β2 subtypes regulate activity in anxiety- and fear-related circuits and have thus been linked to anxiety- and fear-like outputs (Picciotto, 2003; Mineur et al., 2016; Jiang et al., 2016; Wilson and Fadel, 2017; Salas et al., 2003). In humans, individuals report the self-medication of nicotine, an exogenous agonist to nAChRs, for ameliorating anxiety symptoms (Moylan et al., 2012). There is an association between smoking history and mood disorders such as anxiety disorders (Mouro Ferraz Lima et al., 2023) implicating nicotine and nAChRs in anxiety regulation. Further, there is an association between CHRNA4, the gene that encodes the α4 subunit of nAChRs and negative emotionality (Markett et al., 2011). As such, a modulatory protein of nAChRs, such as LYNX2, may be a candidate to consider in anxiety regulation in humans (Palumbo and Miwa, 2023; Miwa et al., 2019; Pisapati et al., 2021).

To assess the translational potential of the *LYNX2* (*LYPD1*) gene for affective disorders, we investigated *LYNX2* and its possible association with anxiety in the human population. We chose to analyze the trait and state components of anxiety in this study as both are represented by rodent behavior paradigms that have been shown to be altered in mice lacking *Lynx2* (Tekinay et al., 2009). Trait anxiety is considered an individual’s tendency toward anxiety. In mice, it is thought that basal anxiety-like behavior can be assessed in the light–dark paradigm, which is elevated in mice lacking *Lynx2*, and is representative of human trait anxiety (Smelser and Baltes, 2001; O’Leary et al., 2013). In humans, trait anxiety manifests as the kind of persistent anxiety that characterizes Generalized Anxiety Disorder (GAD). State anxiety, on the other hand, is acquired through the experience of specific external events/stimuli via learned associations, and in mice can be assessed through paradigms such as fear conditioning/extinction (Hascoët et al., 2001; Elwood et al., 2012; O’Leary et al., 2013). State anxiety can manifest as a phobia, which is also a fear response to a specific stimulus. Here, we identified a subpopulation of individuals harboring a protein coding mutation in *LYNX2*, which we find is associated with heightened self-reported levels representative of both state and trait anxiety. We demonstrate in single-molecule binding studies that this mutation causes a change in the interaction strength of the LYNX2 protein and nAChR complexes.

## 2 Materials and methods

### 2.1 Psychological testing

All tests and the biodemographic/individual information have been approved by the Lehigh University Institutional Review Board. Recruitment was done widely, with the majority of participants being Lehigh University students, but also included students, faculty and administrators at Lehigh and other institutions. All subjects were adults over the age of 18. We collected background information from our participants, which included the following: sex, age, professional/career goals, hobbies/hobby frequency, mental health diagnoses, highest degree attained, medications, smoking habits, alterations related to identity, and previous experience with cognitive/anxiety experiences. This comprehensive data helped us gain insights into the diverse experiences and backgrounds of our participants, allowing us to consider other factors that may contribute to the relevance of our study (Supplementary Table S1). We collected from 624 participants who completed the anxiety questionnaires (STICSA and/or STAI), 179 male participants and 416 female participants, the balance being participants who answered “other” or did not answer that question. The ratio of male and female participants reflected the demographics of the recruitment pool, who were largely Lehigh University natural science students. The average age was 20.7 +/− 0.28 years and ranged from 18 to 63 with a median age of 20. All data underwent a double coding system to ensure anonymity, confidentiality and security of data. Collection of data and samples occurred in batches of roughly 50–100 participants, and samples from each batch were analyzed by different sets of researchers in different locations to reduce the likelihood that a detected SNP allele was a result of a spurious event such as contamination.

The State–Trait Inventory for Cognitive and Somatic Anxiety (STICSA) (Ree et al., 2008) and State–Trait Anxiety Inventory (STAI) (Spielberger et al., 1983) were used to examine human anxiety levels. These tests assess subjects’ chronic and current anxiety levels based on self-reports. The STICSA takes cognitive and somatic anxiety dimensions into account. Both tests provide summarized state and trait anxiety scores calculated from frequency ratings that subjects provide for a variety of anxiety-related symptoms. Participants are asked to report on the anxiety symptoms they are experiencing at the time of the test (state) as well as the anxiety symptoms they experience more generally, outside the bounds of the test and throughout their daily lives (trait). The STAI furthermore allows for a standardized interpretation taking into account inherent anxiety level differences in different populations (based on age, student status, etc.). In addition to anxiety tests, a battery of creativity tests which measure convergent and divergent thinking, including the Creativity Behavioral Inventory (CBI), Remote Associates Test (RAT), and Alternative Uses Task (AUT), were used to examine human creativity due to previous reports of the correlation between creativity and mental illness (Andreasen, 1987; Runco and Richards, 1997; Kyaga et al., 2011; Kyaga et al., 2012). The researchers analyzing the psychology data were different from those performing DNA analysis, to keep the information about SNP carriers blind to those analyzing psychological data.

### 2.2 DNA analysis

Genomic DNA was extracted from saliva samples collected from each participant during psychological testing. Saliva and cheek cells were deposited into conical tubes by the participants after rinsing with a 0.9% saline solution for 60 s. The saline and saliva samples were kept on ice at 4°C for up to one week. A portion of the saliva samples were used for genomic DNA extraction with Chelex resin (BioRad 1421253) following the manufacturer’s recommendations within a week of collection. The remainder of the saliva-saline samples were moved into long-term storage at −20° or − 80°C. Exons 3–4 of the *LYNX2* gene representing the mature protein coding sequence were amplified with PCR primers (Table 1) 100–200 bp outside of the exon/intron boundaries. Primers were identified by comparing isoform cDNA sequences from NCBI (GeneBank name: *LYPD1*, chromosomal location 2q21.2). Successful PCR reactions were purified using ExoSap (Affymetrix cat no.: 78200) or by gel excision of visualized bands and gel extraction (GeneJET Gel Extraction Kit, ThermoFisher, K0691). Purified PCR products were sent to GenScript USA for Sanger sequencing. Chromatogram analyses of sequence files were performed using DNA Baser Sequence Assembler software to generate contigs of overlapping sequences under the high-quality option. If a contig did not form under high quality, the results were discarded, and PCR was performed again for high-quality sequence results.

**Table 1:** PCR Primers used for targeted sequencing of *LYNX2*.

	Exon 2 (ENSE00003691134)	Exon 3 (ENSE00001384648)
Forward	5’-GTGGGATGGTCGTGATTTCCG-3’	5’-GGGACAGCTATTCTTTTGCC-3’
Reverse	5’-CGATAAATTACCGGGGGAGTG-3’	5’-CTCCTTGTCCTACCACCAC-3’

Alternative allele detection was conducted using manual identification of the chromatograms by more than one individual to verify alternative allele t identification. Alignment to the reference genome (GRCh38) was done using Molecular Evolutionary Genetics Analysis (MEGA) software. We performed 2x sequence coverage on all samples using both a forward and reverse sequencing primer, analyzing in large batches of samples, with further in-depth (4–6x) sequence coverage for select samples. Samples from individuals with variant alleles and a representative number of reference samples were further confirmed through extra rounds of amplification and sequencing, as well as re-extraction of DNA, each performed by unique researchers. Eight of the nine identified SNPs were in dbSNP.^1^ The ninth SNP was not previously reported in dbSNP, Gnomad^2^, or Bravo^3^ at the time of publication.

### 2.3 Computational modeling and simulation of *LYNX2*/α7 nAChR complexes

The structure of two adjacent α7 subunits (α7:α7) was taken from the Protein Data Bank (PDB) entry 3SQ6 (Li et al., 2011). The α7 subunits have a closed state conformation of loop C. We obtained 350 α7:α7 snapshots with apo-state loop C conformations from a 10 ns molecular dynamics (MD) simulation by applying a weak positional restraint to glutamate185 Cα atom in the loop C.

The three-dimensional (3D) structure of LYNX2 was predicted from the sequence using the standalone I-TASSER suite (Yang et al., 2015) by specifying a LYNX1 PDB structure (PDB entry 2 L03) (Lyukmanova et al., 2011) as a template. We obtained 100 energy-minimized snapshots from a 5 ns MD simulation of the LYNX2 model and then identified the best snapshot among them by evaluating the quality of each structure using ModFOLD (Maghrabi and McGuffin, 2017). For the best snapshot, we sampled thermally acceptable 50 conformations for LYNX2 loop I using the Backrub application in Rosetta (Smith and Kortemme, 2008).

A low-resolution structure model of α7 nAChR in complex with LYNX2 was generated based on our LYNX1/α4:α4 complex model (Nissen et al., 2018). The α7 subtype was chosen because a homomeric subtype was more tractable to model than heteromeric nAChRs α4β2 and α4β2 subtypes, to which LYNX2 also binds. A set of LYNX2/α7:α7 complex models was built by mapping each α7 subunit (in the ensemble of 350 α7:α7 conformations) and each *LYNX2* (in the ensemble of 50 LYNX2 conformations) onto α4:α4 and LYNX1 in the LYNX1/α4:α4 model, respectively, thus generating 350 × 50 combinations. For each complex, *LYNX2* was moved outwards from α7:α7 by ≤2 Å using an interval of 1 Å along a vector defining the geometric centers of two protein binding surfaces to find a complex structure with the minimum number of bad (energetically unfavorable) contacts between α7:α7 and LYNX2, which was determined as the final model of α7 nAChR in complex with LYNX2 (Supplementary Figure S1).

The LYNX2/α7:α7 complex model was subjected to 135 ns MD simulations to obtain a refined complex model. 5 ns simulations with or without weak positional restraints were iterated during the first 35 ns simulations. The positional restraint was applied to the Q39 Cα atoms of LYNX2, aiming at maintaining LYNX2 loop II in the ligand-binding pocket under α7:α7 loop C by gradually reducing the force constant. We also applied positional restraints to prevent α7:α7 from dissociating; no restraints were applied to loop B, loop C and β8–β9 loop in α7:α7, allowing for conformational flexibility, as they involve protein–protein interactions. After the 35 ns simulation, we removed the positional restraints to LYNX2 and performed additional 100 ns simulations for equilibration.

The Q61H mutation of the full-length *LYNX2* gene (the nascent polypeptide before cleavage of the signal sequence) is at amino acid 39 in the fully-processed mature LYNX2 protein and will be referred to as Q39H. To generate the LYNX2 Q39H alternative allele, we computationally replaced LYNX2 Q39 with histidine in the α7:α7/ LYNX2 complex structure refined by MD simulations (Supplementary Figure S1). We carried out 400 ns MD simulation without any positional restraints to LYNX2 for three independent replicates (MD-1, MD-2, and MD-3) of α7:α7 in complex with native LYNX2 and its Q39H alternative allele. The convergence of the simulations was assessed by measuring root-mean-square deviation (RMSD) of LYNX2 in terms of simulation time, indicating that MD-3 s of each system show good convergence (Supplementary Figure S1C). The trajectories of these well-converged replicates were then analyzed to calculate nonbonded interaction (van der Waals and electrostatic) potential energies between the entire α7:α7 interface and LYNX2. Note that, to calculate the RMSD, we first aligned α7:α7 of each MD snapshot to the initial structure (i.e., the structure before the MD simulations) and then calculated the RMSD of the entire LYNX2 Cα atoms relative to its initial structure. Therefore, the calculated RMSD includes both the LYNX2 conformational changes and the changes of LYNX2 positions relative to α7:α7. Given the flexibility of α7:α7/LYNX2 interactions, RMSD values above 5 Å appear to be reasonable and α7:α7 and LYNX2 interact strongly as shown in Supplementary Figure S1C (the interaction energy time series) (Dong et al., 2020).

**Figure 1:**
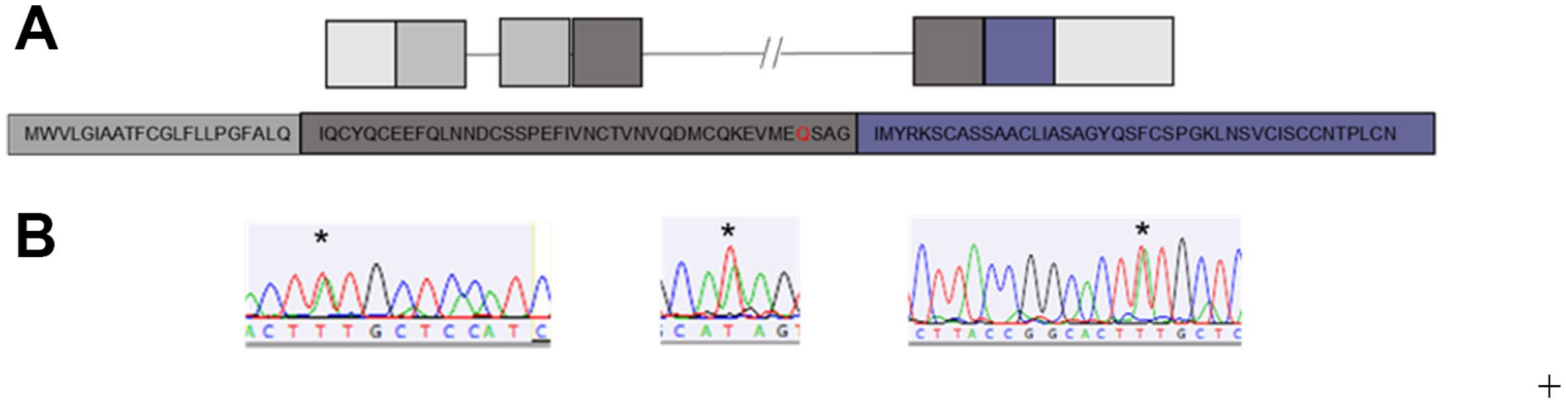
Wild-type Human *LYNX2* gene mutants. **(A)** Schematic of the wild-type human *LYNX2* gene and corresponding amino acid sequence. Light grey = UTR, dark grey = signal sequence, black = exon 3, blue = exon 4. **(B)** Representative chromatographs from mutant individuals, stars show heterozygous peak.

All MD simulations were prepared through CHARMM-GUI (Jo et al., 2008; Lee et al., 2016) and performed with explicit water molecules and 150 mM NaCl using CHARMM36m force field (Huang et al., 2017) and NAMD software (Phillips et al., 2005) at 300 K.

### 2.4 Proteins

Recombinant hexahistidine-tagged human *LYNX2* gene was made in the pcDNA3.1 plasmid, expressed in HEK 293 T cells and purified from culture supernatant using Ni-NTA chromatography. The expression and purification of the protein were confirmed by SDS-PAGE and Western blot using an anti-his-HRP antibody.

### 2.5 Protein immobilization

Purified recombinant LYNX2 was attached to an atomic force microscopy (AFM) cantilever by covalently cross-linking the two using a heterobifunctional PEG linker (Ebner et al., 2007). The AFM cantilevers (MLCT: Bruker Nano, Camarillo, CA) were first silanized with 3-aminopropyltriethoxysilane (APTES) using a gas phase coating method (Wildling et al., 2011). An acetal-PEG27-NHS linker was then attached to the silanized cantilevers in chloroform via its N-Hydroxysuccnimide (NHS) group and then rinsed and dried under nitrogen gas. Next, the acetal group of the PEG linker was converted to an aldehyde group using a 1% citric acid solution vol/vol, and 1 μM of LYNX2 was then added to the solution, which coupled to the activated PEG linker. After quenching the free aldehyde group with ethanolamine, the cantilevers were stored in TBS buffer (50 mM Tris, 0.15 M NaCl, pH 7.4) until experimental use.

### 2.6 AFM apparatus

All single-molecule force measurements were conducted using a custom-designed AFM that employs a single-axis piezoelectric translator equipped with a strain gauge (Physik Instrumente, Waldbronn, Germany) to control the absolute position of the AFM cantilever (Chen and Moy, 2002; Zhang et al., 2002; Zhang et al., 2004; Fu et al., 2015). The deflection of the cantilever was monitored optically using an inverted optical system attached to the AFM. A focused laser spot from a fiber-coupled diode laser was reflected off the back of the cantilever onto a two-segment photodiode to monitor the cantilever deflection. The photodiode signal was then pre-amplified, digitized and processed by a computer. The force apparatus was suspended inside a refrigerator housing to reduce both mechanical and thermal instabilities.

The human α7 nAChR receptor was expressed on SH-EP1 cells by transfection of pCEP4 vector containing the cDNA of the α7 receptor. The stably transfected SH-EP1-hα7-nAChR cell line was previously engineered and characterized as described in Zhao et al. (2003). The transfected cells were maintained in media supplemented with 0.13 mg/mL hygromycin B. The cells were plated on 35 mm cell culture dish 24 h prior to AFM force measurements.

### 2.7 AFM force measurements of individual LYNX2/α7 nAChR interactions

**Figure 2:**
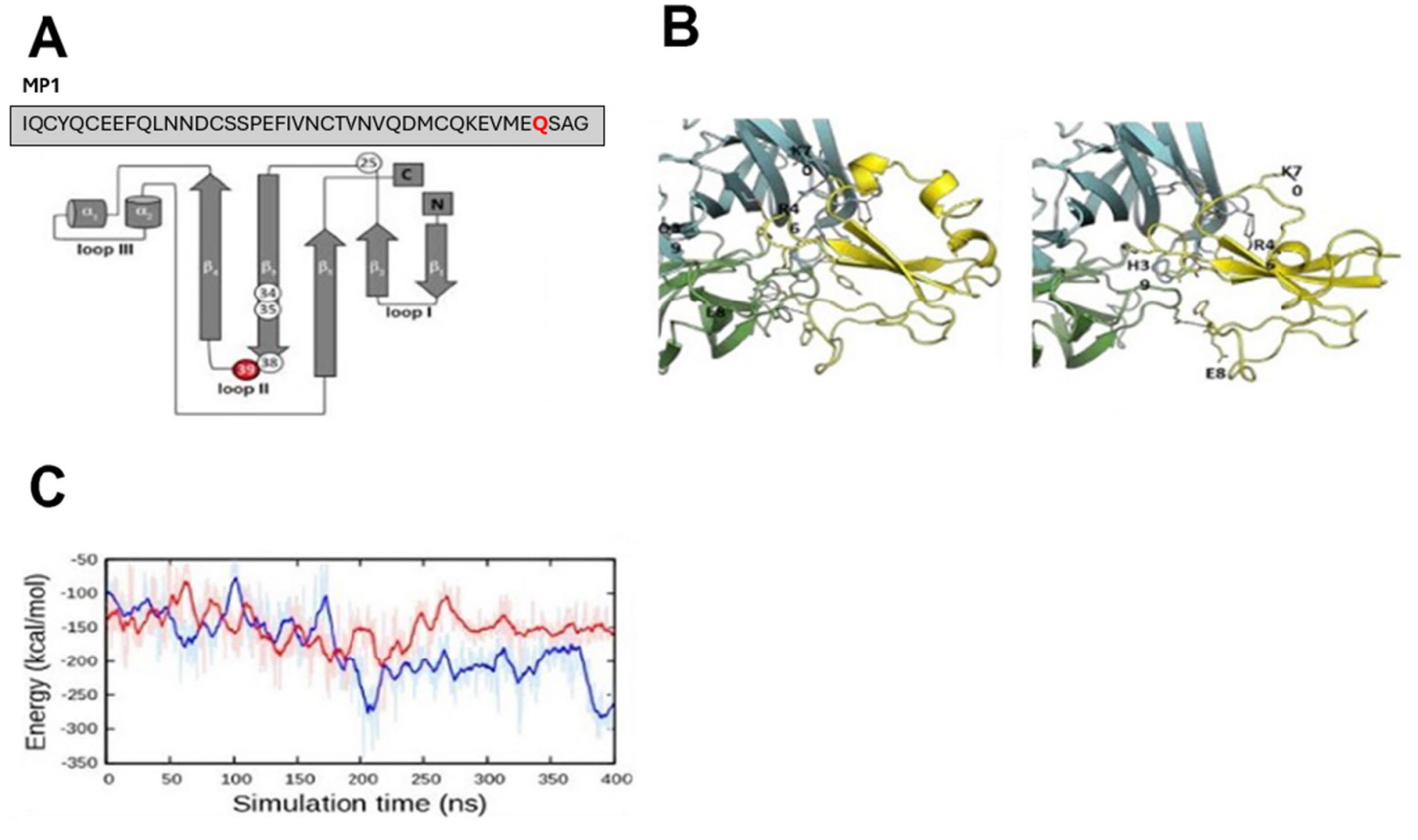
Protein modeling of *LYNX2* and *LYNX2*/α7 nAChR interaction. **(A)** Human *LYNX2* protein structure schematic. **(B)** Left: Structural details of α7:α7 in complex with native *LYNX2*. The last 400-ns simulation snapshot was used for this structural illustration. Two α7 subunits are in green and cyan. *LYNX2* is colored yellow. Hydrogen bonds between α7:α7 and *LYNX2* are highlighted using orange dotted lines. Right: Structural details of α7:α7 in complex with mutant *LYNX2*. The last 400 ns simulation snapshot was used for this structural illustration. Two α7 subunits are in green and cyan. *LYNX2* is colored yellow. Hydrogen bonds between α7:α7 and *LYNX2* are highlighted using orange dotted lines. **(C)** Interaction energy between α7:α7 and *LYNX2* during the simulations.

The AFM force measurements were performed on an apparatus designed for operation in the force spectroscopy mode (Zhang et al., 2002; Zhang et al., 2004; Zhang et al., 2009; Fu et al., 2015). Using a piezoelectric translator, the LYNX2-functionalized cantilever tip was lowered onto a live SH-EP cell expressing α7 nAChRs (Figure 2A). Tip-cell contact may (or may not) lead to LYNX2/α7 nAChR interactions. The binding interaction was then determined from the deflection of the cantilever via a position-sensitive two-segment photodiode. To calibrate the cantilever (320 μm long by 22 μm wide triangle), the spring constant at the tip was characterized via thermally induced fluctuations (Hutter and Bechhoefer, 1993). The spring constants (9.7 ± 1.9 pN/nm) of the calibrated cantilevers agreed with the values specified by the manufacturer.

Except for the adhesion specificity test (Supplementary Figure S1D), the contact time and indentation force between the cantilever and the sample were minimized to obtain measurements of the unitary unbinding force. An adhesion frequency of ~30% in the force measurements ensured that there was a > 83% probability that the adhesion event was mediated by a single LYNX2/α7 nAChR bond (Evans, 2001). AFM measurements were collected at cantilever retraction speeds ranging from 0.1 to 10 μm/s in order to achieve the desired loading rate (~30–4,000 pN/s). All measurements were conducted at 25°C in the cell culture medium. Loading rates were determined directly from the force-extension data by multiplying the system spring constant (Figure 2A) of the unbinding pulling trace by and the retraction speed of the cantilever.

### 2.8 Dynamic force spectroscopy

The Bell-Evans model, a theory to determine energy landscape properties, describes the influence of an external force on the rate of bond dissociation (Bell, 1978; Evans and Ritchie, 1997). According to this model, a pulling force, *f*, distorts the intermolecular potential of a receptor-ligand complex, leading to a lower activation energy and an increase in the dissociation rate *k(f)* or a decrease in the lifetime *t*(*f*) as follows:


(1) $$k\left(f\right)=\frac{1}{t\left(f\right)}=k^{0}e^{\left(\frac{f\gamma }{k_{B}T}\right)}$$

where k^0^ is the dissociation rate constant in the absence of a pulling force, *γ* is the width of the activation barrier, or the position along the reaction coordinate relative to the bound state, *T* is the absolute temperature, and *k_B_* is the Boltzmann constant. For a constant loading rate *r*_f_, the probability density for the unbinding of the complex as a function of the pulling force *f* is given by:


(2) $$P\left(f\right)=k^{0}e^{\left(\frac{\gamma f}{k_{B}T}\right)}e^{\left\{\frac{k^{0}k_{B}T}{\gamma r_{f}}-\left[1-e^{\left(\frac{f\gamma }{k_{B}T}\right)}\right]\right\}}$$

with the most probable unbinding force f*.


(3) $$f^{*}=\frac{k_{B}T}{\gamma }\ln\left(\frac{\gamma }{k^{0}k_{B}T}\right)+\frac{k_{B}T}{\gamma }\ln\left(r_{f}\right)$$

Hence, the Bell-Evans model predicts that the most probable unbinding force f* is a linear function of the logarithm of the loading rate. Experimentally, f* was determined from the mode of the unbinding force histograms. The Bell-Evans model parameters were determined by fitting Equation 3 to the plot of f* versus ln(*r_f_*).

### 2.9 Statistical analysis (methods)

The STAI (State–Trait Anxiety Inventory) was administered in this study because it distinguishes clearly between “state” anxiety, which refers to anxiety being experienced at the time of assessment, and “trait” anxiety, which predicts the degree to which a person is prone to generalized anxiety. To assess participants’ (*n* = 624) state and trait anxieties, the complete STAI test was administered to two cohorts, which we will refer to as the “discovery cohort” and the “replication cohort.”

Half of the STAI addresses state anxiety, and participants are asked to answer assessment items intuitively and in accordance with their present feelings. This part of the test includes items such as “I feel calm,” “I am tense,” and “I feel satisfied,” and participants are asked to characterize their agreement with the statement on a scale of 1–4. 1 corresponds to the answer “Not at all,” and 4 to the answer “Very much so.”

The other half addresses trait anxiety, in which participants are asked to answer in accordance with their feelings in general, and beyond the scope of the time in which the test was taken and into their daily lives. This part of the test includes items such as “I feel pleasant,” “I feel satisfied with myself,” and “I lack self-confidence,” and participants are asked to characterize their agreement with the statement on the same scale of 1–4.

To score the STAI, we added the scoring weights of each question in accordance with the official manual. For the statements which indicate more anxiety (e.g., “I feel tense”), a participant scoring of 4 corresponds to an item weight of 4, and a participant scoring of 1 corresponds to an item weight of 1. For the statements that, inversely, indicate less anxiety (e.g., “I feel calm”), a participant scoring of 4 corresponds to an item weight of 1, and a participant scoring of 1 indicates an item weight of 4, such that the increased weight of an item corresponds to more anxiety-expressing responses.

The scoring of the STICSA was comparable to that of the STAI. The STICSA also makes the distinction between state and trait anxieties, and total scores were calculated by the summation of the items’ weights, in accordance with the STICSA manual. STAI responses were analyzed according to the STAI manual.

Statistical analysis involved comparing scores of human *LYNX2* mutant populations to scores of the *LYNX2* WT, or reference, population. This was done with a two-tailed Fisher’s Exact Test, or an independent two-tailed *t*-test, to determine whether there existed a statistically significant difference between the means of the groups. In some tests, we combined state and trait results of the STICSA and STAI to produce a “total score,” and in others, we made the distinction between state and trait in order to test them separately. In performing a Fisher’s Exact Test to compare scores between human participants with and without the *LYNX2* Q39H mutation, we chose a threshold score of 43 for the STICSA test based on literature determining that it was the score that could predict incidence of an anxiety disorder in individuals (Van Dam et al., 2013). For the STAI, we chose a threshold score of 45 as a score that has been used in literature to represent “high anxiety” (Kayikcioglu et al., 2017).

The Creative Behavioral Inventory, CBI, was analyzed with a Fisher Exact Test comparing the average total CBI score of those with the Q39H mutation and the reference group. The CBI total score is determined by summing the individual scores of its components, which are Literature, Music, Crafts, Art, Math & Science, and Performing Arts. Average scores for mutant and reference groups were found and compared, according to each population’s size.

### 2.10 AFM

For each pulling speed, over 1,000 force curves were recorded, which yielded 80 to 350 unbinding forces. Curve fitting was performed using IGOR Pro or Origin software by minimizing the chi-square statistic for the optimal fit. Unless otherwise stated, the data is reported as the mean and the standard error of the estimate. Statistical analyses between groups were performed using an unpaired *t*-test or ANOVA, with a *p*-value less than 0.05 considered to be statistically significant.

## 3 Results

### 3.1 *LYNX2* variants are associated with altered anxiety phenotypes

To assess if *LYNX2* has an impact on human anxiety, we investigated whether there is a correlation between variation in the *LYNX2* gene and self-reported anxiety in a population of 624 participants. The average age of our available population (M = 20 +/− 0.28 years) was below the average age of clinical diagnosis of anxiety disorders at the time of testing (M = 30, Gonçalves and Byrne, 2012; Vaingankar et al., 2013; Lijster et al., 2017). We used two established tests of self-reported anxiety for phenotyping: the State–Trait Inventory for Cognitive and Somatic Anxiety (STICSA) and the State–Trait Anxiety Inventory (STAI). The STICSA was chosen because it is designed to discriminate between symptoms of anxiety and depression. These two disorders are often comorbid, which can make interpretation of genetic risk for anxiety difficult. The STAI was chosen because it can discriminate between the state and trait components of anxiety (Oei et al., 1990; Grӧs et al., 2007; Elwood et al., 2012; Van Dam et al., 2013).

**Figure 3:**
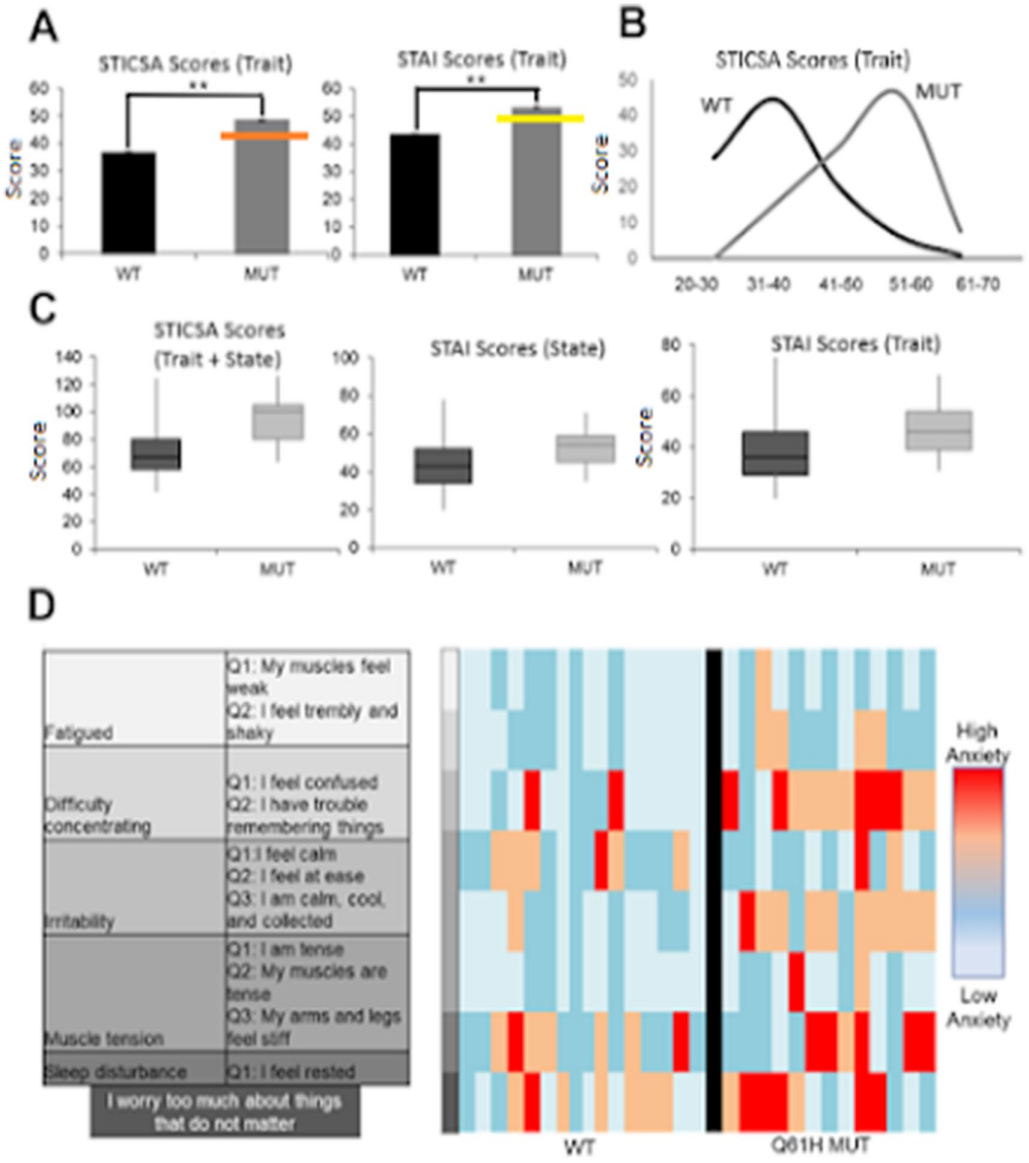
Individuals harboring *LYNX2* mutations have increased anxiety levels. **(A)** Wildtype (WT) and mutant (MUT) anxiety scores, STICSA trait (left) and STAI trait (right). Orange line is the level used to diagnose generalized anxiety disorder and the yellow line are scores at the level used to diagnose panic disorder population (STICSA: *p* < 1.0 × 10–4, *n* = 558 *WT*; 14 *MUT*; STAI: *p* = 4.0 × 10–4, *n* = 553 *WT*; 14 *MUT* Error bars represent ±SEM). **(B)** Population histogram of STICSA trait, scores binned in 10 point increments. **(C)** Population box plot comparisons for STICSA total score (left), STAI state (middle), STAI trait (right). **(D)** DSM GAD criteria and corresponding questions from surveys (left), Monte Carlo WT and Q39H question responses (right). In the case of sleep disturbance, “I feel rested,” low scores indicated disturbance and thus were converted to the same continuum.

After extracting DNA and performing targeted Sanger sequencing, we detected SNPs in nine different positions across the mature protein coding sequence of *LYNX2* (Figure 3A). Eight of the nine SNPs were previously reported in dbSNP, and consisted of a mix of synonymous, missense, and frameshift mutations (Table 2). We did not find enough participants harboring these eight mutations to draw conclusive results about how they function in anxiety. One SNP, however, was not previously reported: (Tables 2, 3) a C → T missense variant resulting in a glutamine (Q) to histidine (H) substitution at amino acid 61 of the full-length protein, and amino acid 39 of the mature protein. We will refer to this variant as Q39H. This was the most common SNP variant in our population, occurring in 14/624 (2.2%) of participants, and was exclusively heterozygous. The Q39H variant was found across different batches of samples throughout the course of the study (Supplementary Table S1). None of the nine variants had been previously associated with anxiety or had pre-existing reports of clinical significance.

**Table 2:** Representative mutations found in this population along with anxiety scores.

GRCh37/hg19	Amino acid	Number of participants	STICSA	STICSA	STAI	STAI
Nucleotide position	Consequence	Found in study	State	Trait	State	Trait
WT	–	–	33.9	36.6	37.9	43.2
133426094	I23M	1	37	48	34	54
133426070	F31F	2	33, 37	44, 33	37, 38	44, 32
133426058	N35N	1	34	43	42	53
133426040	E41E	1	33	26	34	51
133426034	I43I	2	37, 29	38, 33	41, 40	43, 41
133426019	V48V	1	36	36	42	50
133425980	Q61H	14	45.9	48.4	46.9	54.0
133425984	E60V	2	28, −	33, −	33, −	35, −
13340373	Frameshift	1	51	68	45	53

Amino acid positions reference the full length protein. Note that some of the mutations are synonymous mutations and do not change the amino acid identity at that position.

**Table 3:** Cohort information.

		Discovery Cohort			Replication Cohort	
Total (*n*)		140			484	
Gender		56 M/84 F			12 M/332 F	
	WT	Mut	WT vs. MUT	WT	Mut	WT vs. MUT
*n*	131	9		469	15	
Mutant info					9-Q61H	
		5-Q61H			1-E60V	
		1-V48V			2-I43I	
		1-E41E			1-N35N	
		2-F31F			1-I23M	
					1-Frameshift	
STICSA Total Score +/− SEM*	68.69 +/− 1.36	95.8 +/− 8.01	*p* = 0.013; Cohen’s *d* = 1.64	71.09 +/− 1.04	91.778 +/− 6.184	*p* = 0.005; Cohen’s d = 1.34
STAI Total Score +/− SEM*	76.85 +/− 1.59	101.6 +/− 9.18	*p* = 0.026; Cohen’s *d* = 1.31	82.76 +/− 1.30	99.0 +/− 6.53	*p* = 0.019; Cohen’s d = 0.82

General information and human anxiety test scores from a discovery and replication cohort. Scores represent the combined “at the moment” and general test scores, and were analyzed by *t*-test. Amino acid positions reference the full-length protein.

As a group, individuals harboring any alternative SNP allele in *LYNX2* reported significantly higher STICSA and STAI scores than those with a reference allele (*p* = 0.005; Cohen’s d = 1.34, Table 3) (Figure 1B). The individuals with the Q39H mutation, in particular, were associated with significantly increased anxiety scores comparable to those clinically diagnosed with an anxiety disorder. Including anxiety, panic attacks, and PTSD. In the STICSA test (Figure 3), the Q39H group was significantly different from the reference group in all measured anxiety tests, with a general value (representative of trait anxiety) of 48.43 +/− 2.11, and the reference group 36.60 +/− 0.40 (*p* < 1.0 × 10^−4^), and “at the moment” value of 45.86 +/− 2.50 compared to the reference group of 33.92 +/− 0.40 (*p* < 1.0 × 10^−4^). In the STAI test, the Q39H group was also significantly different from the reference group, with a “general” value (representative of trait anxiety) of 54.07 +/−2.79, and the reference group 43.28 +/− 0.49 (*p* = 4.0 × 10^−4^), and “at the moment” value (representative of state anxiety) of 46.93 +/− 2.79 compared to the reference group of 37.94 +/− 0.49 (*p* = 0.0281) (Table 2; Figure 3). We found two individuals harboring a synonymous mutation at the same location (nucleotides encoding amino acid 39 of the mature protein and amino acid 61 of full length *LYNX2*) with anxiety levels similar to the reference group. This suggests that only the nonsynonymous mutations confer a functional consequence.

**Figure 4:**
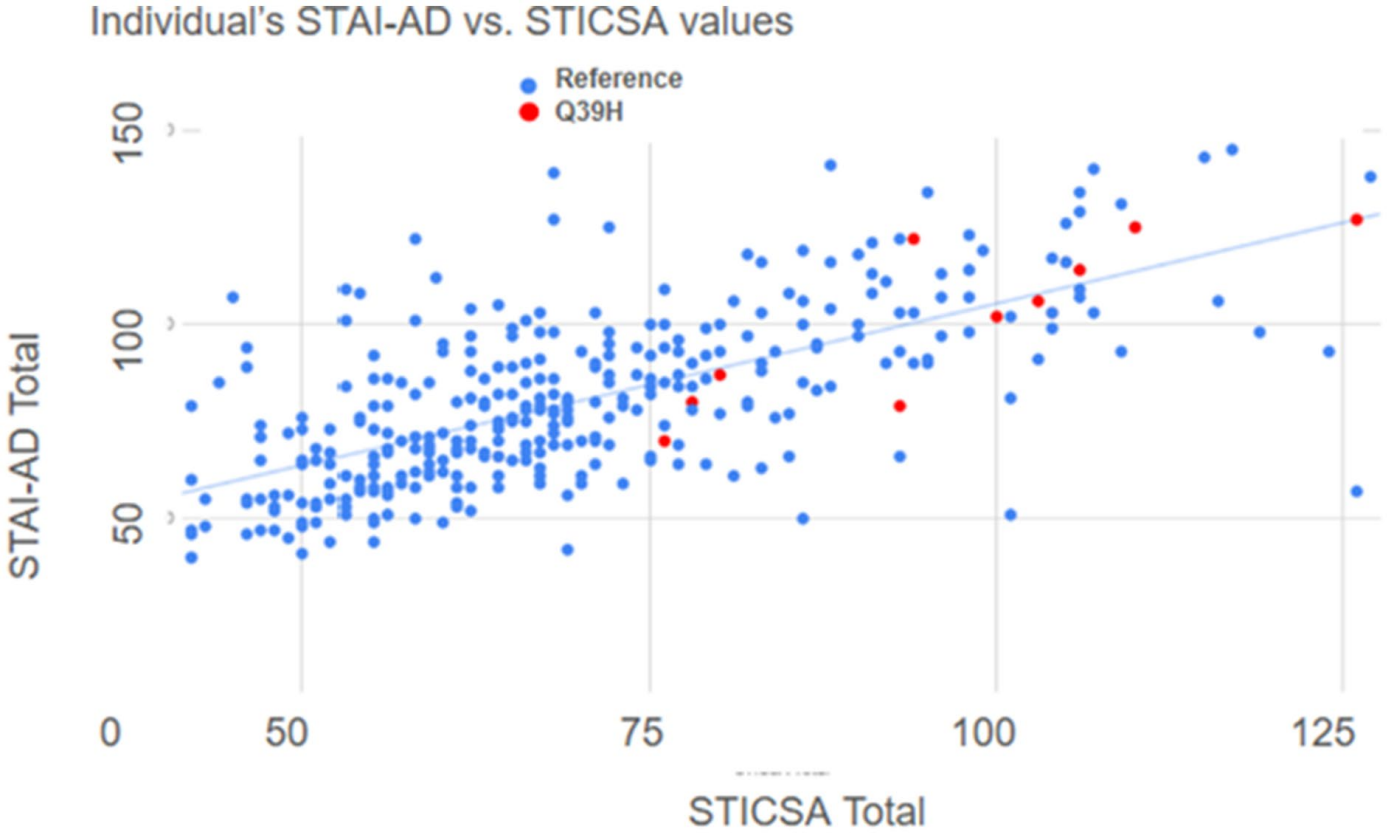
Correlation between STICSA and STAI values by individual. Scatter plot of anxiety scores; STICSA values combining both “at the moment” plus general values of that test, on the x-axis, plotted against the combined STAI values, “at the moment” plus general values, on the y axis. Each dot represents a different individual. Blue represents individuals with the WT *LYNX2* gene and red represents those harboring the Q39H *LYNX2* mutation.

We further analyzed the anxiety test results by comparing answers from questions that matched the Diagnostic and Statistical Manual of Mental Disorders (DSM-5) criteria for Generalized Anxiety Disorder (GAD), an anxiety disorder reflective of increased trait anxiety (Chambers et al., 2004). We identified 11 questions that matched diagnosis symptoms. In these DSM-aligned questions, the Q39H group answers reflected significantly higher anxiety scores (Figure 3D). These data suggest a relationship between the *LYNX2* mutation Q39H and heightened anxiety levels. The individuals with the Q39H mutation in *LYNX2* also had anxiety scores comparable to those of individuals with diagnosed panic disorder (Van dam et al., 2013). 61.5% of individuals harboring the Q39H had high anxiety levels, above the levels for GAD, whereas in the reference group, 15.9% of respondents exhibited such levels. There was good concordance in the entire dataset between the STICSA and STAI values including the reference group and the Q39H carriers (Figure 4). This suggests a functional consequence of the mutation in elevating anxiety levels, indicating its potential role in predisposing individuals to heightened anxiety traits.

**Figure 5:**
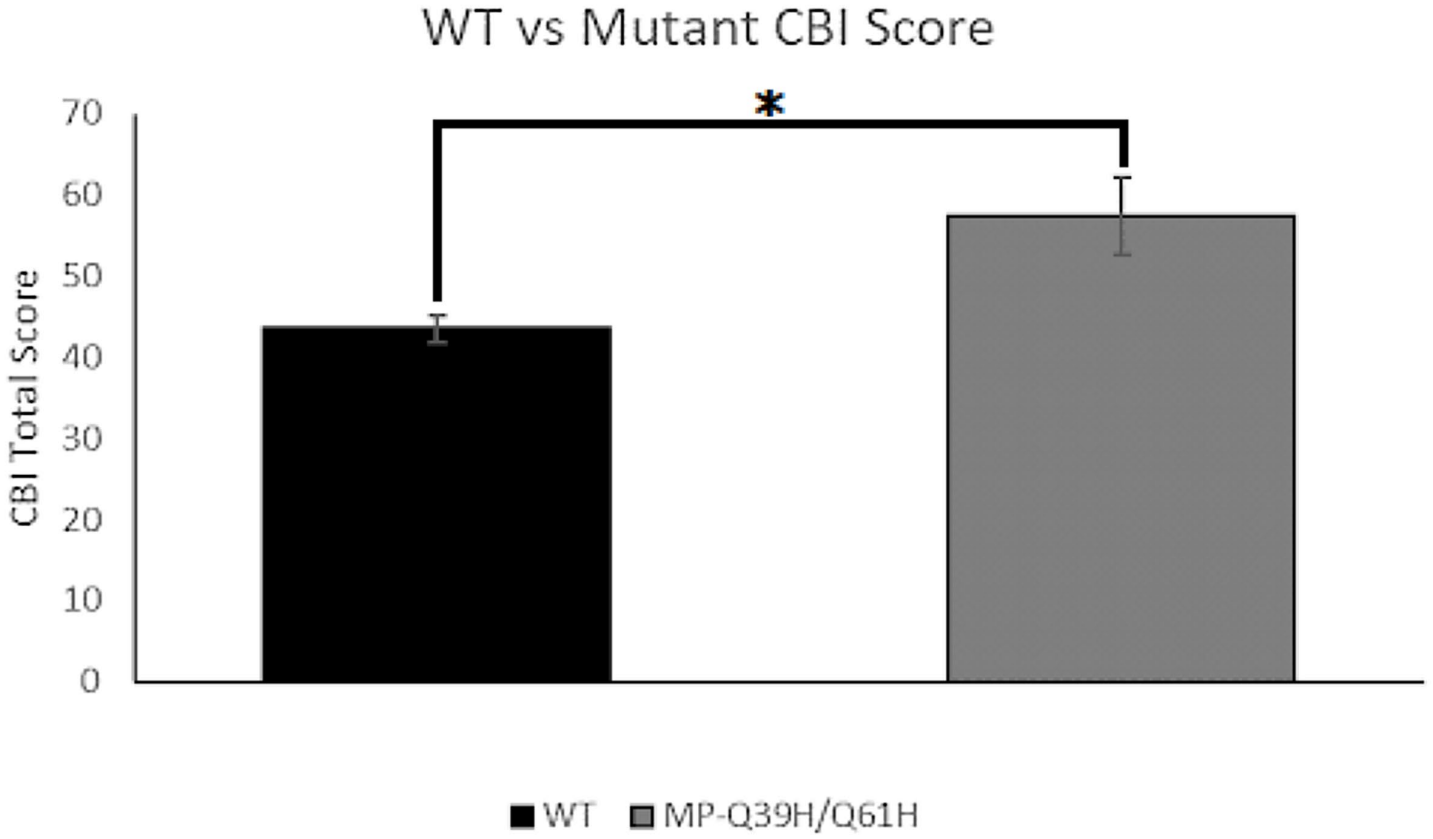
Creative behavioral inventory (CBI) score comparison. Average CBI score for wildtype (WT) vs. Q39H (*n* = 206 *WT*; *n* = 9 *Q39H*). Data were analyzed using a Fisher Exact Test with a threshold CBI score of 40, in which data were organized into a category of a score either <40 or ≥ 40 (*p* = 0.0447).

To explore a previously discovered link between creativity and heightened trait anxiety levels, we performed and analyzed the results of the Creative Behavioral Inventory (CBI), taking interest in the comparison of scores between those harboring the Q39H mutation and those not (Figure 5). We found there to be a significant difference between the average scores of the groups, with a Q39H group score of 30.35 +/− 4.79 and a reference group score of 43.46 +/− 1.62 (*p* = 0.45). This provides evidence for a link between the Q39H mutation and creativity.

### 3.2 Mutated human LYNX2 protein binds with less affinity to *α*7 nAChRs

To study the possible effect of the most common Q39H variant on nAChR binding, we performed computational modeling and binding studies with the human LYNX2 protein and a nAChR subtype. The Q39H mutation is at amino acid 39 in the mature protein (Figure 2A). In the mature protein the 39th amino acid mutation site is at the tip of loop II (Figure 2A), a region associated with nAChR binding similar to three-looped nAChR-binding proteins like α-bungarotoxin (Tsetlin, 2014) and LYNX1 (Lyukmanova et al., 2013).

To examine the possible effect of the mutation at the molecular level, we performed computational modeling and simulations of LYNX2 binding to the α7:α7 interfaces of α7 nAChR (Figure 2), a common nAChR subtype expressed in anxiety pathways of rodents and shown to be critical in regulating amygdala excitability (Klein and Yakel, 2006; Pidoplichko et al., 2013; Almeida-Suhett et al., 2014; Jiang et al., 2016). We chose the α7 subtype to model because a homomeric subtype was more tractable to model than heteromeric nAChRs α4β2 and α4β2 subtypes. A low-resolution structural model of the α7 nAChR in complex with LYNX2 was generated based on our LYNX1/α4:α4 complex model (Nissen et al., 2018). A set of LYNX2/α7:α7 complex models was built by mapping each α7 subunit (in the ensemble of 350 α7:α7 conformations) and each LYNX2 (in the ensemble of 50 LYNX2 conformations) onto α4:α4 and LYNX1 in the LYNX1/α4:α4 model, respectively, thus generating 350 × 50 combinations. From this we selected the complex structure with the minimal number of energetically unfavorable contacts as the final model (Supplementary Figure S1).

The modeling indicates that the wild-type LYNX2 forms a complex with the α7:α7 subunit interface through multiple salt bridges with E8, R46 and K70 of LYNX2 (Figure 2B). However, in the Q39H LYNX2 variant, the LYNX2 and α7:α7 subunit interface salt bridges are established only by R46 of LYNX2 (Figure 2B). Further, three residues in the loop II of WT LYNX2, including Q39, form additional hydrogen bonds with α7:α7. These hydrogen bonds are, however, not observed in the α7:α7 complex with the Q39H variant LYNX2. We measured intermolecular interaction energy between α7:α7 and LYNX2 during the 400 ns molecular dynamics (MD) simulations (Figure 2C). The results indicate a more favorable interaction of WT *LYNX2* with α7:α7 after 200 ns than the Q39H variant. Therefore, our modeling and simulations suggest that the Q39H LYNX2 variant would have a lower binding affinity to α7-nAChRs and thus suppresses the function of α7-nAChRs less than a WT protein.

**Figure 6:**
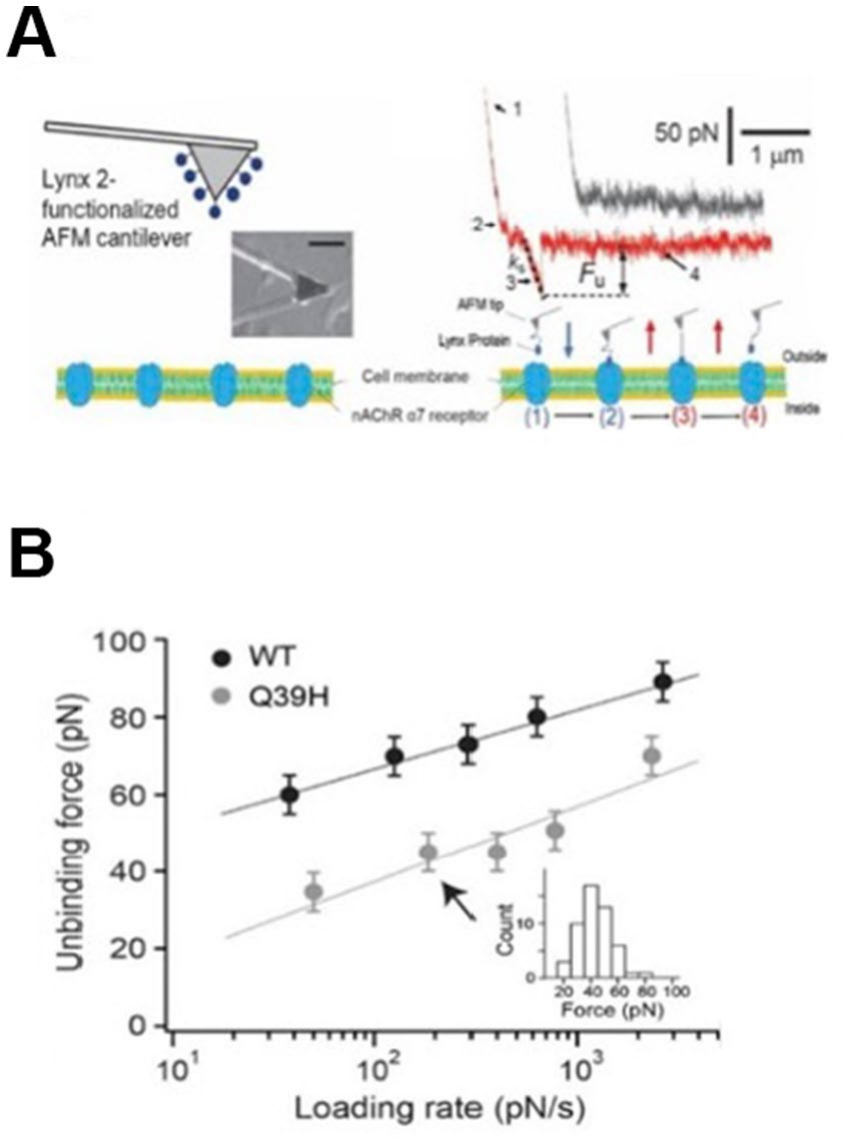
Mutations cause structural changes in the human protein: comparison of binding affinity between native *LYNX2* and the Q39H mutant. **(A)** Micrograph of AFM microcantilever with *LYNX2* above α7/SH-EP cells and sample AFM pulling traces. The black (upper) trace shows no interaction, and the red (lower) trace shows the rupture force of a single *LYNX2*/α7 complex. Unbinding force = Fu. ks is the system spring constant and was derived from the slope of the force-displacement trace, retraction rate 1.8 μm/s. Four AFM stages of stretching and rupturing a single ligand-receptor complex (lower panel). **(B)** Dynamic force spectrum (plot of most probable unbinding force/loading rate) of the *LYNX2*/α7 interactions. Unbinding forces at different loading rates were plotted as histograms. The peak of each histogram (i.e., the most probable unbinding force) was plotted against the loading rate; uncertainty in the unbinding forces is shown as half of the bin width. The linear fits were obtained using Equation 3 (Chen and Moy, 2002; Zhang et al., 2009).

To further gain insight into the molecular consequences of naturally occurring variants underlying the LYNX2/nAChR interactions, we applied single-molecule atomic force spectroscopy (AFM) to study the reaction kinetics between LYNX2 and α7 nAChRs at the single-molecule level. For these studies, purified recombinant LYNX2 was covalently attached to an AFM cantilever and a SH-EP1 mammalian cell line stably expressing concatemeric α7 nAChRs (Pisapati et al., 2021) were used (Figure 6A). LYNX2/α7 nAChR interactions were analyzed by dynamic force spectroscopy (DFS), the plot of most probable unbinding force (Figure 6B, Equations 1–3). The unbinding force of the LYNX2/α7 nAChR interaction increased with the logarithm of the loading rate, ranging from 60 to 90 pN over a loading rate of 30–4,000 pN/s, respectively (Figure 6B, Equations 1–3). The *LYNX2* variant with the Q39H mutation found in the human population resulted in significantly decreased unbinding force under all tested loading rates tested. The mutation shortened the lifetime of the interaction by 180-fold (from 1,470 to 8.1 s). This result indicates that this human mutation in LYNX2 weakens the physical association with α7 nAChRs *in vitro*.

## 4 Discussion

In this study, we have identified a correlation between the genomic presence of a *LYNX2* variant, Q39H, and clinically relevant levels of anxiety in humans. The *LYNX2* mutation we found in our population confers reduced binding affinity of the LYNX2 protein to its target, nAChRs. Taken together, reduced binding of the LYNX2 protein that results from the Q39H mutation, in some cases, could result in abnormal anxiety levels in individuals harboring the mutation.

We found that the most common human *LYNX2* variant, Q39H, was associated with responses that aligned with the DSM criteria for anxiety disorder diagnoses, GAD and panic disorder. The behavioral expression of fear results from a relative weighting of key components of the circuits underlying fear behavior. Murine *Lynx2* is expressed in several of these key regions in mice, including the prefrontal cortex and amygdala (Tekinay et al., 2009; Dessaud et al., 2006). The amygdala is an important structure in basal fear and anxiety behavior in humans (Martin et al., 2009; LeDoux, 2011), whereas the prefrontal cortex can have roles in assessing and modifying fear-based learning association (Fanselow and LeDoux, 1999; Sierra-Mercado et al., 2010; Ottaviani et al., 2012; Lebois et al., 2019; Morgan et al., 1993; Milad and Quirk, 2002; Quirk et al., 2000; Milad et al., 2006; Sierra-Mercado et al., 2010; Amano et al., 2010; Do-Monte et al., 2015; Mukherjee et al., 2018). Any factor which alters the normal activity in these circuits (amygdala, prefrontal cortex and limbic cortex) may influence anxiety levels. nAChRs have been implicated in the regulation of anxiety (Mineur et al., 2023). A mutation in a nAChR modulator such as *LYNX2*, that disrupts the normal binding capability, protein levels, or activity has the potential to play a role in the regulation of anxiety by altering nAChR function.

The Q39H mutation led to a *LYNX2* variant with decreased binding affinity to α7 nAChRs, which to our knowledge, is the first demonstration of a naturally occurring mutation in the human LYNX2 protein with a defined biological or biophysical consequence (Anderson et al., 2020). Furthermore, the binding data provide compelling evidence that the human *LYNX2* gene operates in a similar manner as mouse *Lynx2* via its modulation of nAChRs. In *Lynx2* null mutant mice, nAChR responses are hyperactive, responding with hypersensitivity to agonist and slower desensitization rates. This perturbation is accompanied by elevations in both basal and state anxiety-like behavior (Tekinay et al., 2009). In the case of the human Q39H carriers, we suggest that LYNX2 protein is expressed but does not have the binding, and in turn inhibitory capability, of wild-type LYNX2, the consequence of which is nAChR hypersensitivity and elevated anxiety levels.

Although the heightened anxiety scores seen in the variant/alternate allele subpopulations match those of diagnosed population levels, our population does not report a high percentage of clinical diagnosis, likely due to the age of participants in our study (*M* = 20 years of age). Although the predicted age of onset of anxiety disorders was found to average 21.3 years of age in a meta-analysis, anxiety disorders at the time of this study were often not clinically diagnosed until 30 years of age (Gonçalves and Byrne, 2012; Vaingankar et al., 2013; Lijster et al., 2017). In addition, median age of onset varies greatly depending on the disorder; while the GAD median age of onset is 30, social anxiety and specific phobia diagnosis typically occur earlier in life, most often during adolescence. The majority of our cohort being students, they face a composite of stressors different from that of older populations. As such, it cannot be predicted if a similar incidence would be found in older individuals. Because of the select nature of the cohort tested, investigation of this variant in the general population including a greater number of older individuals would be helpful in evaluating the persistence of the effect of such a mutation on anxiety levels. Not all the carriers in our cohort exhibited higher than normal levels of anxiety (>60% of Q39H carriers exhibited elevated anxiety levels, compared to <20% in the reference group), underscoring the complex nature of anxiety and as would be expected, other factors than a single gene would play into the behavioral expression of anxiety (Parodi et al., 2022). The Q39H allele is novel and not found in databases, so caution must be applied to the interpretation of these findings. A new mutation at the same nucleotide position was entered into a human database subsequent to the time period of data collection indicating that novel mutations in the *LYNX2* gene may still be forthcoming.

It would be interesting to test if the Q39H mutation occurred at a higher rate in the GAD or panic disorder population as compared to a general population. Identifying individuals with susceptibility toward anxiety in such a way could enable earlier preventative measures, such as monitoring, stress management techniques or psychotherapy. This would be significant as unregulated anxiety can lead, not only to an anxiety disorder, but also to depression (Maron and Nutt, 2017). There is potential for *LYNX2* to have utility as a genetic screen to aid in identification of susceptible individuals before anxiety becomes severe or an entrenched disorder. Such a genetic screen could be an advance given the paucity of biomarkers for mental illnesses. Recently, there have been reports that expression differences of *LYNX2* may help to differentiate Bipolar Disorder from Schizophrenia and MDD (Shen et al., 2024). Natural variation in the *Lynx2* gene, however, has been associated only in animal strains that exhibit altered fear conditioning (Parker et al., 2012), or with processes outside of the nervous system, such as cancer and skin abnormalities (Sugimori et al., 2024; Song et al., 2024).

Though we tested binding of the LYNX2 protein to α7 nAChRs by AFM, we did not test for binding of other known targets of LYNX2, such as α3β4 and α4β2 subtypes, and it would be worthwhile to investigate binding of Q39H protein on those subtypes as well. It is worth noting the potential relevance of LYNX2’s interaction with α3β4 nAChRs, a subtype that has been implicated in anxiety regulation in brain regions such as the medial habenula and the interpeduncular nucleus (IPN) (McLaughlin et al., 2017; Molas et al., 2017). LYNX2 has been found to bind with higher affinity to a3β4 nAChRs as compared to α7 nAChRs (Pisapati et al., 2021), and thus α3β4 nAChRs are a probable target of the effect of LYNX2 action. The medial habenula/IPN has been implicated mediating anxiety induced by nicotine withdrawal (Molas et al., 2017), and is a region that highly expresses α3 and β4 nAChR subunits (Grady et al., 2009). The cohort examined in this study contained a low level of those self-reporting nicotine intake. Further studies on a smoking population or those subject to nicotine dependence may bring insights into the effect of human *LYNX2* and α3β4 nAChRs. In-depth studies on α3β4 might be fruitful by performing nicotine withdrawal tests in the *Lynx2* KO mouse model and focusing on the medial habenula/IPN pathway. The α7 nAChR subtype has been shown to play a role in anxiety-like regulation of animal behavior (O’Connor et al., 2024, Mineur et al., 2023). Further, CHRNA7, the gene that encodes α7 nAChRs, has been associated with anxiety and depressive disorders in clinical trials (Gillentine et al., 2018). Polymorphisms in the human CHRNA4 gene, which encodes the α4 nAChR subunit, has been linked to anxiety and emotional lability (Markett et al., 2011), and α4β2 nAChRs have been linked to anxiety in a number of studies (McGranahan et al., 2011; Mineur et al., 2023). The nAChR target of LYNX2 has been reported for α4β2, α7, and α3β4 nAChR, but the interaction of LYNX2 on other subtypes, such as α4β4, α6- or α5-containing nAChRs, etc. to our knowledge has not been tested as yet. Much further work will be necessary to understand the binding of LYNX2 on different nAChR subtypes and their effects on different CNS processes.

*LYNX2* expression is not confined to the anxiety-related circuit, and thus some of the elevated anxiety phenotype could be amplified or blunted by effects from other regions or influence other processes that were not measured here. Another potential explanation for the lower percentage of carriers exhibiting low levels of anxiety diagnoses, could be rooted in the fact that this variant/alternate allele subpopulation also displays heightened scores used to measure human creativity, indicating a potential, previously suggested, association between creativity and anxiety (Kyaga et al., 2012). Studies show that creative outlets may be used to temporarily decrease anxiety supporting this potential correlation (Müller et al., 2008; King et al., 2013). Further, human studies have shown a relationship between enhanced associative learning and creative thought (Beaty and Kenett, 2023). While not directly translatable, *Lynx2* KO mice demonstrate elevated associative learning in a fear-conditioning paradigm (Tekinay et al., 2009). Since these mice also demonstrate elevated basal levels of anxiety-like behavior, this phenotype cannot be directly interpreted as enhanced learning. This mouse model does, however, demonstrate heightened responses to a nAChR agonist in the prefrontal cortex, measured by EPSC rate, suggesting a physiological consequence and possible alterations in circuit function (Tekinay et al., 2009) and behaviors (Sherafat et al., 2021) outside the amygdala. Further characterization of the allele in humans has the potential to shed light on the neural processes enabling novel or creative thought and/or provide a biological explanation for the correlation between creative individuals and mental illness (Andreasen, 1987; Runco and Richards, 1997; Kyaga et al., 2011; Kyaga et al., 2012).

Our data suggest a potential diagnostic avenue for those suffering from an anxiety disorder by identifying individuals harboring a deleterious *LYNX2* mutation that may sensitize nAChRs within specific neural circuits. Despite compelling evidence of the role of nAChRs in affective disorders, large clinical trials targeting nAChRs have failed, demonstrating that targeting of nAChRs in a general population is not generally effective (Vieta et al., 2013). Smaller studies with specific inclusion criteria have shown success clinically (Phillips et al., 2005), raising the possibility that nAChR targeting could be a more effective treatment within a specific subpopulation. Genetic discriminators may be one such defining marker. A genetically identified subpopulation harboring this *LYNX2* mutation, entered into clinical trials may lead to better outcomes for nAChR candidates than when tested in a general population of individuals with better moderated nAChR responses. Alternatively, it may identify those who may be responsive to already approved medications such as the nAChR blocker mecamylamine (Bacher et al., 2009). These results have implications for the much-needed early identification of those with a genetic predisposition to anxiety, and hence for the possible prevention of the development of an anxiety disorder.

## Data Availability

The original contributions presented in the study are included in the article/Supplementary materials, further inquiries can be directed to the corresponding author.

## Footnotes

^1^ https://www.ncbi.nlm.nih.gov/snp/

^2^ https://gnomad.broadinstitute.org/

^3^ https://bravo.sph.umich.edu/freeze8/hg38/
