# Supplementary figures and images for: A novel anxiety-associated SNP identified in LYNX2 (LYPD1) is associated with decreased protein binding to nicotinic acetylcholine receptors
Source: Front Behav Neurosci. 2024 Dec 23;18:1347543. doi: 10.3389/fnbeh.2024.1347543 (PMC11702307; doi:10.3389/fnbeh.2024.1347543)

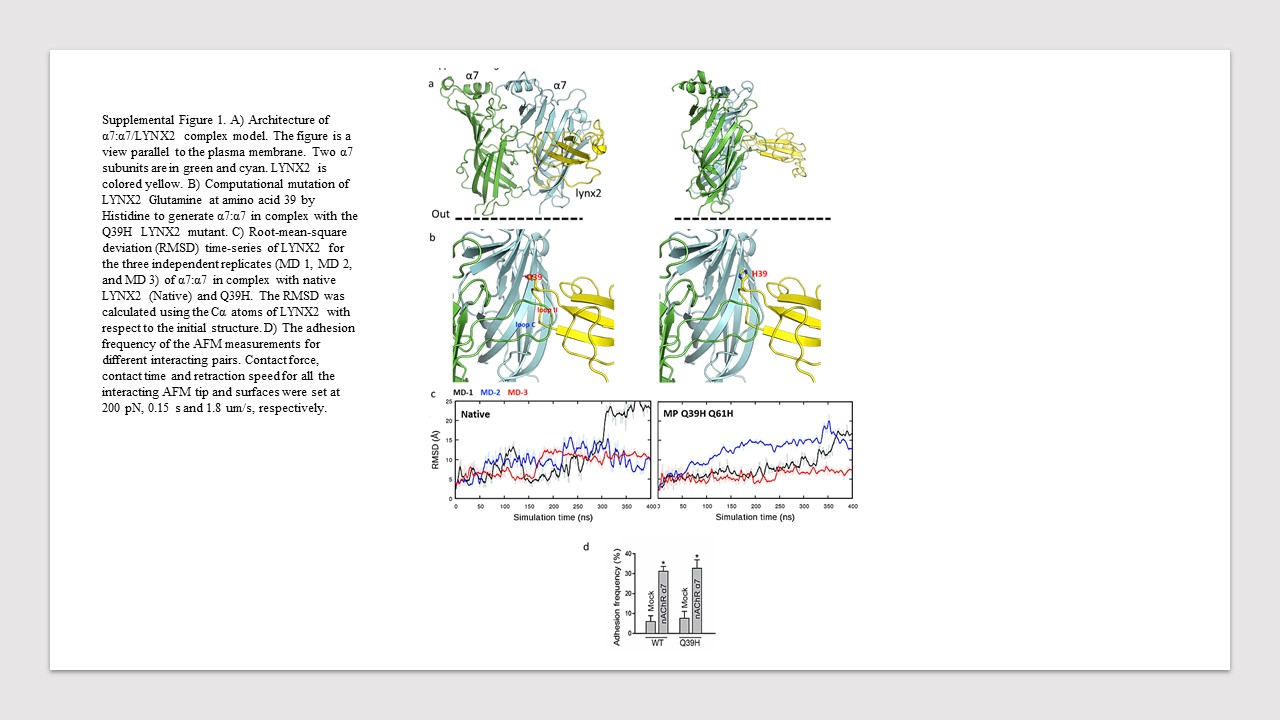

Supplement: Supplementary file 1 [file Image_1.jpg]
