# Supplementary material for: A novel anxiety-associated SNP identified in LYNX2 (LYPD1) is associated with decreased protein binding to nicotinic acetylcholine receptors
Source: Front Behav Neurosci. 2024 Dec 23;18:1347543. doi: 10.3389/fnbeh.2024.1347543 (PMC11702307; doi:10.3389/fnbeh.2024.1347543)
Supplement: Supplementary file 2 [file Table_1.pdf]

Supplemental Table 1 Biodemographic information of the SNP allele (Q39H) carriers

The results show questions that we found particularly noteworthy, as they offer valuable insights into the demographics and characteristics of our study population. Medications listed were those to address neuropsychiatric disorders only. Both sex and gender identity were questioned, and gender identification was the same as sex assigned at birth for all SNP carriers who answered. Smoking refers to smoking at least one pack of nicotine-containing products. Sample and psychological data collection occurred in batches of 50-100 participants, and the batch number for that participant is listed. Blanks represent no answer by the participant.

Carrier	Gender	Age	Batch	Diagnoses	Smoking	Medication
1	female	20	1	no	no	ADHD
2			1			no
3	female	18	2	no	no	
4	female	19	2	no		no
5	female	19	2	no		no
6	female	19	3			no
7	female	22	5		no	
8	female	21	5	anxiety, depression	yes	anxiety
8	female	20	5		no	
10	female	18	5		no	
11	female	19	6	no	yes	
12	female	18	6	no	yes	
13	female	21	8	n/a	no	
14	female	19	8		yes	
